# 4,4′-Ethyl­enedipyridinium bis­(3,4,5-trihy­droxy­benzoate) sesquihydrate

**DOI:** 10.1107/S1600536810027169

**Published:** 2010-07-17

**Authors:** Fwu Ming Shen, Shie Fu Lush

**Affiliations:** a Department of Biotechnology, Yuanpei University, No.306, Yuanpei St, HsinChu, Taiwan 30015; b Department of Medical Laboratory Science Biotechnology, Yuanpei University, HsinChu, Taiwan 30015

## Abstract

The asymmetric unit of the title compound, C_12_H_14_N_2_^2+^·2C_7_H_5_O_5_^−^·1.5H_2_O, contains two 4,4′-ethyl­enedipyridinium cations, four gallate anions and three water mol­ecules. In the 4,4′-ethyl­enedipyridinium cations, the dihedral angles between the pyridinium rings are 4.3 (3) and 18.6 (3)°. Extensive classical N—H⋯O and O—H⋯O hydrogen bonding and weak C—H⋯O hydrogen bonding and C—H⋯π inter­actions are present in the crystal structure. π–π stacking is also observed, the centroid–centroid separations between the benzene and pyridine rings being 3.611 (3), 3.448 (3) and 3.536 (3) Å.

## Related literature

For related structures, see: Bebout & Pagola (2009 ▶); Li & Guo (2007 ▶); Okabe *et al.* (2001 ▶). For the biological activity of gallic acid (3,4,5-trihy­droxy­benzoic acid) and its derivatives, see: Fukumoto & Mazza (2000 ▶).
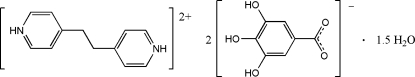


## Experimental

### 

#### Crystal data

C_12_H_14_N_2_^2+^·2C_7_H_5_O_5_^−^·1.5H_2_O
*M* *_r_* = 551.50
Triclinic, 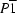

*a* = 9.4445 (19) Å
*b* = 15.331 (3) Å
*c* = 18.307 (4) Å
α = 109.88 (3)°
β = 93.68 (3)°
γ = 92.90 (3)°
*V* = 2480.2 (10) Å^3^
*Z* = 4
Mo *K*α radiation
μ = 0.12 mm^−1^
*T* = 295 K
0.32 × 0.30 × 0.22 mm

#### Data collection

Nonius KappaCCD diffractometer
Absorption correction: multi-scan (*SCALEPACK*; Otwinowski & Minor, 1997 ▶) *T*_min_ = 0.928, *T*_max_ = 1.016
26683 measured reflections
8520 independent reflections
5004 reflections with *I* > 2σ(*I*)
*R*_int_ = 0.104

#### Refinement

*R*[*F* ^2^ > 2σ(*F* ^2^)] = 0.099
*wR*(*F* ^2^) = 0.221
*S* = 1.35
8520 reflections
713 parameters
H-atom parameters constrained
Δρ_max_ = 0.47 e Å^−3^
Δρ_min_ = −0.34 e Å^−3^

### 

Data collection: *COLLECT* (Nonius, 2000 ▶); cell refinement: *SCALEPACK* (Otwinowski & Minor, 1997 ▶); data reduction: *DENZO* (Otwinowski & Minor, 1997 ▶) and *SCALEPACK*; program(s) used to solve structure: *SHELXS97* (Sheldrick, 2008 ▶); program(s) used to refine structure: *SHELXL97* (Sheldrick, 2008 ▶); molecular graphics: *PLATON* (Spek, 2009 ▶); software used to prepare material for publication: *PLATON*.

## Supplementary Material

Crystal structure: contains datablocks global, I. DOI: 10.1107/S1600536810027169/xu2790sup1.cif

Structure factors: contains datablocks I. DOI: 10.1107/S1600536810027169/xu2790Isup2.hkl

Additional supplementary materials:  crystallographic information; 3D view; checkCIF report

## Figures and Tables

**Table 1:** Hydrogen-bond geometry (Å, °) *Cg*6 and *Cg*8 are the centroids of the C33–C38 and C47–C52 rings, respectively.

*D*—H⋯*A*	*D*—H	H⋯*A*	*D*⋯*A*	*D*—H⋯*A*
N1—H1*A*⋯O13^i^	0.86	1.98	2.793 (5)	158
N1—H1*A*⋯O14^i^	0.86	2.32	2.914 (5)	127
N2—H2*A*⋯O11	0.86	1.74	2.571 (5)	163
N2—H2*A*⋯O12	0.86	2.60	3.297 (5)	139
N3—H3*B*⋯O1^ii^	0.86	1.67	2.514 (5)	166
N4—H4*C*⋯O3^iii^	0.86	1.97	2.804 (5)	165
N4—H4*C*⋯O4^iii^	0.86	2.47	3.026 (5)	123
O3—H3*A*⋯O7	0.82	1.83	2.652 (4)	175
O4—H4*B*⋯O16^iv^	0.82	2.06	2.804 (4)	151
O5—H5*B*⋯O6^v^	0.82	1.81	2.634 (5)	180
O8—H8*A*⋯O21	0.82	1.90	2.715 (4)	175
O9—H9*B*⋯O15^vi^	0.82	2.47	3.153 (4)	141
O10—H10*B*⋯O15^vi^	0.82	2.12	2.812 (5)	142
O13—H13*A*⋯O17^vii^	0.82	1.75	2.569 (4)	172
O14—H14*A*⋯O15	0.82	2.32	2.729 (4)	111
O15—H15*A*⋯O16	0.82	1.89	2.713 (4)	179
O18—H18*A*⋯O23	0.82	2.25	2.623 (5)	108
O20—H20*A*⋯O21	0.82	2.59	3.185 (5)	131
O21—H21*A*⋯O2	0.86	1.92	2.680 (5)	148
O21—H21*B*⋯O19^ii^	0.86	2.32	3.163 (4)	165
O22—H22*A*⋯O9^viii^	0.86	1.86	2.713 (4)	170
O22—H22*B*⋯O11	0.86	1.99	2.855 (5)	179
O23—H23*A*⋯O12^v^	0.86	1.80	2.637 (5)	166
O23—H23*B*⋯O22^ix^	0.86	1.92	2.781 (5)	174
C11—H11*A*⋯O23	0.93	2.53	3.293 (7)	139
C34—H34*A*⋯O20	0.93	2.40	3.265 (6)	154
C4—H4*A*⋯*Cg*6^ii^	0.93	2.81	3.571 (5)	140
C16—H16*A*⋯*Cg*8	0.93	2.91	3.737 (5)	149

Symmetry codes: (i) 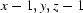; (ii) 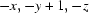; (iii) 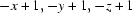; (iv) 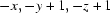; (v) 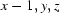; (vi) 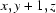; (vii) 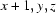; (viii) 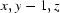; (ix) 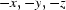.

**Fig. 1.**
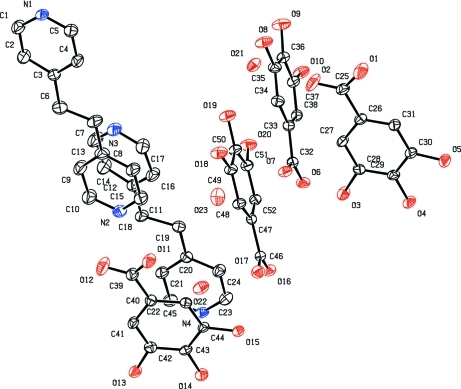
View of the title compound with the atom numbering scheme. Displacement ellipsoids for non-H atoms are drawn at the 30% probability level.

**Fig. 2.**
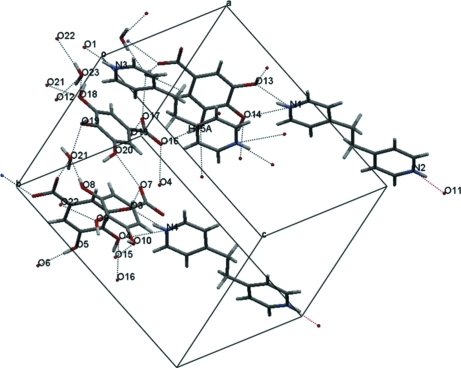
The molecular packing for the title compound. Hydrogen-bonding associations are shown as dotted lines.

## supplementary crystallographic information

### Comment

Gallic acid, 3,4,5-trihydroxybenzoic acid, and derivatives have been found to be antitumor and antioxidative activities (Fukumoto & Mazza,2000). Their related structures have been reported (Okabe *et al.*,2001; Li & Guo, 2007; Bebout & Pagola, 2009).

The crystal structure of the hydrated proton-transfer compound of gallic acid with 4,4'-ethylenedipyridine, 2(C_12_ H_14_ N_22_^+2^), 4(C_7_ H_5_ O_5_^-^). 3(H_2_O), contains two 4,4'-ethylenedipyridinium cations, four gallate anions and three water molecules in the asymmetry unit (shown as Fig.1). In the two 4,4'-ethylenedipyridinium cations, the dihedral angles between the pyridyl rings are 4.3 (3) and 18.6 (3) °. Three gallate anions have intramolecular hydrogen bonding. The hydroxyl H atoms bound to O4, O9, O10 and O14 (donors) form intramolecular hydrogen bonds to O5, O10, O9 and O15 (acceptors), respectively. The 4,4'-ethylenedipyridinium cations is linked by N—H···O hydrogen bonds to adjacent gallate anions, forming linear hydrogen bonded chains parallel to [1 0 1]. The structure exhibits a three dimension hydrogen-bonding network by involving *O*—*H*···*O*(carboxylate) interactions. Intermolecular N—H···O, O—H···O and C—H···O hydrogen bondings are observed in the crystal structure (shown as Table 1, Fig. 2).

On the other hand, π–π ring stacking interactions are between benzene and pyridinium rings with centroid-centroid distances in the range 3.446 (3)\sim 3.612 (3) Å, the shortest distance between *Cg*4(N4/C20—C24)···*Cg*5(C26—C31) is 3.446 (3)Å and dihedral angle between two rings is 3.6 (2)° [symmetry code: 1+*X*,Y,*Z*]. In addition, C—H···π interactions C4—H4···*Cg*6(C33—C38), C16—H16A···*Cg*8(C47—C52); full details and symmetry codes are given in Table 1) are also present.

### Experimental

The gallic acid (171.0 mg, 1.0 mmol) and 4,4'-ethylenedipyridine (184 mg, 1.0 mmol) were dissolved in 20 ml e thanol-water(2:1), the solution was refluxed for 30 min. The filtered solution was transferred to a 25 ml tube after one week at room temperature and pale pink transparent crystals formed (yield 48.19%).

### Refinement

Water H atoms were located in a difference Fourier map and refined with the distances constraints of O—H = 0.86 Å, U_iso_(H) = 1.5U_eq_(O). Other H atoms were positioned geometrically with O—H = 0.82, N—H = 0.86, C—H = 0.93 (aromatic) and 0.97 Å (methylene), and were refined using a riding model with U_iso_(H) = 1.2U_eq_(C,N) and 1.5U_eq_(O).

### Crystal data

**Table:**

C_12_H_14_N_2_^2+^·2C_7_H_5_O_5_^−^·1.5H_2_O	*Z* = 4
*M**_r_* = 551.50	*F*(000) = 1156
Triclinic, *P*1	*D*_x_ = 1.477 Mg m^−^^3^
Hall symbol: -P 1	Mo *K*α radiation, λ = 0.71073 Å
*a* = 9.4445 (19) Å	Cell parameters from 6466 reflections
*b* = 15.331 (3) Å	θ = 2.0–25.4°
*c* = 18.307 (4) Å	µ = 0.12 mm^−^^1^
α = 109.88 (3)°	*T* = 295 K
β = 93.68 (3)°	Prism, pale pink
γ = 92.90 (3)°	0.32 × 0.30 × 0.22 mm
*V* = 2480.2 (10) Å^3^	

### Data collection

**Table:**

Nonius KappaCCD diffractometer	8520 independent reflections
Radiation source: fine-focus sealed tube	5004 reflections with *I* > 2σ(*I*)
graphite	*R*_int_ = 0.104
Detector resolution: 9 pixels mm^-1^	θ_max_ = 25.0°, θ_min_ = 2.3°
ω/2θ scans	*h* = −10→11
Absorption correction: multi-scan (*SCALEPACK*; Otwinowski & Minor, 1997)	*k* = −18→18
*T*_min_ = 0.928, *T*_max_ = 1.016	*l* = −21→21
26683 measured reflections	

### Refinement

**Table:**

Refinement on *F*^2^	Secondary atom site location: difference Fourier map
Least-squares matrix: full	Hydrogen site location: inferred from neighbouring sites
*R*[*F*^2^ > 2σ(*F*^2^)] = 0.099	H-atom parameters constrained
*wR*(*F*^2^) = 0.221	*w* = 1/[σ^2^(*F*_o_^2^) + (0.080*P*)^2^] where *P* = (*F*_o_^2^ + 2*F*_c_^2^)/3
*S* = 1.35	(Δ/σ)_max_ < 0.001
8520 reflections	Δρ_max_ = 0.47 e Å^−^^3^
713 parameters	Δρ_min_ = −0.34 e Å^−^^3^
0 restraints	Extinction correction: *SHELXL97* (Sheldrick, 2008), FC^*^=KFC[1+0.001XFC^2^Λ^3^/SIN(2Θ)]^-1/4^
Primary atom site location: structure-invariant direct methods	Extinction coefficient: 0.0046 (11)

### Special details

**Table:**

Geometry. Bond distances, angles *etc*. have been calculated using the rounded fractional coordinates. All su's are estimated from the variances of the (full) variance-covariance matrix. The cell e.s.d.'s are taken into account in the estimation of distances, angles and torsion angles
Refinement. Refinement on *F*^2^ for ALL reflections except those flagged by the user for potential systematic errors. Weighted *R*-factors *wR* and all goodnesses of fit *S* are based on *F*^2^, conventional *R*-factors *R* are based on *F*, with *F* set to zero for negative *F*^2^. The observed criterion of *F*^2^ > σ(*F*^2^) is used only for calculating -*R*-factor-obs *etc*. and is not relevant to the choice of reflections for refinement. *R*-factors based on *F*^2^ are statistically about twice as large as those based on *F*, and *R*-factors based on ALL data will be even larger.

### Fractional atomic coordinates and isotropic or equivalent isotropic displacement parameters (Å^2^)

**Table:**

	*x*	*y*	*z*	*U*_iso_*/*U*_eq_	
N1	0.0070 (4)	0.1378 (3)	−0.5346 (2)	0.0429 (14)	
N2	0.3616 (4)	0.1056 (3)	−0.0603 (2)	0.0511 (16)	
C1	0.1482 (5)	0.1376 (4)	−0.5309 (3)	0.0588 (19)	
C2	0.2202 (5)	0.1285 (4)	−0.4683 (3)	0.059 (2)	
C3	0.1489 (5)	0.1181 (3)	−0.4071 (3)	0.0436 (17)	
C4	0.0033 (5)	0.1187 (4)	−0.4146 (3)	0.0528 (19)	
C5	−0.0657 (5)	0.1285 (4)	−0.4789 (3)	0.0538 (19)	
C6	0.2334 (6)	0.1095 (5)	−0.3369 (3)	0.066 (2)	
C7	0.1507 (6)	0.1160 (4)	−0.2694 (3)	0.061 (2)	
C8	0.2315 (5)	0.1118 (3)	−0.1966 (3)	0.0478 (17)	
C9	0.3767 (6)	0.1152 (4)	−0.1849 (3)	0.063 (2)	
C10	0.4416 (6)	0.1120 (4)	−0.1156 (3)	0.062 (2)	
C11	0.2211 (6)	0.1015 (4)	−0.0708 (3)	0.058 (2)	
C12	0.1541 (6)	0.1044 (4)	−0.1371 (3)	0.0601 (19)	
N3	0.4594 (5)	0.3694 (3)	−0.0725 (2)	0.0559 (18)	
N4	0.8108 (4)	0.3965 (3)	0.4206 (2)	0.0424 (14)	
C13	0.5989 (6)	0.3883 (5)	−0.0571 (3)	0.070 (3)	
C14	0.6672 (5)	0.3919 (4)	0.0115 (3)	0.061 (2)	
C15	0.5926 (5)	0.3742 (3)	0.0680 (3)	0.0408 (17)	
C16	0.4471 (5)	0.3556 (4)	0.0523 (3)	0.0543 (19)	
C17	0.3848 (5)	0.3548 (4)	−0.0184 (3)	0.060 (2)	
C18	0.6711 (5)	0.3755 (4)	0.1427 (2)	0.0446 (19)	
C19	0.5841 (5)	0.3929 (4)	0.2125 (3)	0.0420 (17)	
C20	0.6656 (5)	0.3950 (3)	0.2859 (2)	0.0354 (16)	
C21	0.8103 (5)	0.3868 (3)	0.2908 (3)	0.0452 (17)	
C22	0.8806 (5)	0.3879 (3)	0.3587 (3)	0.0487 (17)	
C23	0.6707 (5)	0.4047 (3)	0.4187 (3)	0.0446 (17)	
C24	0.5966 (5)	0.4043 (3)	0.3523 (3)	0.0425 (17)	
O1	−0.3781 (4)	0.6359 (3)	0.2075 (2)	0.0792 (16)	
O2	−0.1568 (4)	0.6257 (3)	0.1798 (2)	0.0803 (16)	
O3	0.0893 (3)	0.6021 (2)	0.43137 (17)	0.0480 (13)	
O4	−0.1136 (3)	0.6249 (2)	0.52719 (17)	0.0487 (11)	
O5	−0.3839 (3)	0.6427 (3)	0.48367 (18)	0.0563 (14)	
C25	−0.2473 (5)	0.6308 (3)	0.2254 (3)	0.0441 (17)	
C26	−0.2122 (5)	0.6294 (3)	0.3059 (2)	0.0352 (16)	
C27	−0.0736 (5)	0.6189 (3)	0.3292 (2)	0.0380 (17)	
C28	−0.0425 (4)	0.6155 (3)	0.4034 (2)	0.0349 (16)	
C29	−0.1483 (5)	0.6252 (3)	0.4543 (2)	0.0355 (16)	
C30	−0.2869 (5)	0.6346 (3)	0.4293 (2)	0.0382 (17)	
C31	−0.3195 (5)	0.6361 (3)	0.3551 (2)	0.0377 (16)	
O6	0.4085 (3)	0.7394 (2)	0.45724 (16)	0.0445 (11)	
O7	0.2951 (3)	0.6363 (2)	0.35056 (17)	0.0410 (11)	
O8	0.1899 (3)	0.8332 (2)	0.17878 (17)	0.0523 (11)	
O9	0.3017 (4)	1.0079 (2)	0.26833 (18)	0.0576 (14)	
O10	0.4411 (3)	1.0399 (2)	0.41025 (17)	0.0493 (11)	
C32	0.3465 (5)	0.7175 (3)	0.3894 (3)	0.0353 (17)	
C33	0.3355 (4)	0.7930 (3)	0.3552 (2)	0.0332 (14)	
C34	0.2652 (4)	0.7745 (3)	0.2810 (2)	0.0366 (17)	
C35	0.2552 (5)	0.8458 (3)	0.2510 (2)	0.0379 (17)	
C36	0.3112 (5)	0.9355 (3)	0.2946 (3)	0.0367 (17)	
C37	0.3818 (5)	0.9525 (3)	0.3671 (2)	0.0360 (17)	
C38	0.3918 (4)	0.8822 (3)	0.3980 (2)	0.0343 (14)	
O11	0.4349 (4)	0.1139 (3)	0.07976 (17)	0.0547 (13)	
O12	0.6552 (4)	0.1230 (3)	0.0530 (2)	0.0733 (16)	
O13	0.9199 (3)	0.1324 (2)	0.31510 (17)	0.0454 (11)	
O14	0.7270 (3)	0.1783 (2)	0.41445 (16)	0.0447 (11)	
O15	0.4537 (3)	0.2027 (2)	0.37189 (15)	0.0372 (10)	
C39	0.5680 (5)	0.1251 (3)	0.0995 (3)	0.0431 (17)	
C40	0.6112 (5)	0.1418 (3)	0.1840 (2)	0.0355 (16)	
C41	0.7500 (5)	0.1325 (3)	0.2087 (2)	0.0378 (16)	
C42	0.7860 (4)	0.1435 (3)	0.2851 (3)	0.0363 (16)	
C43	0.6847 (5)	0.1665 (3)	0.3391 (2)	0.0329 (16)	
C44	0.5473 (4)	0.1789 (3)	0.3151 (2)	0.0319 (16)	
C45	0.5111 (5)	0.1657 (3)	0.2376 (2)	0.0336 (16)	
O16	0.2520 (3)	0.3050 (2)	0.33605 (17)	0.0447 (11)	
O17	0.1281 (3)	0.1815 (2)	0.25025 (18)	0.0440 (11)	
O18	−0.0104 (4)	0.2787 (2)	0.01976 (17)	0.0511 (11)	
O19	0.0747 (4)	0.4575 (2)	0.06270 (17)	0.0515 (11)	
O20	0.2244 (4)	0.5481 (2)	0.20255 (17)	0.0505 (11)	
C46	0.1795 (5)	0.2653 (3)	0.2719 (3)	0.0340 (17)	
C47	0.1545 (4)	0.3173 (3)	0.2172 (2)	0.0304 (14)	
C48	0.0839 (4)	0.2722 (3)	0.1439 (2)	0.0366 (16)	
C49	0.0586 (4)	0.3185 (3)	0.0922 (2)	0.0353 (16)	
C50	0.1037 (5)	0.4130 (3)	0.1146 (2)	0.0373 (17)	
C51	0.1760 (5)	0.4580 (3)	0.1875 (3)	0.0366 (17)	
C52	0.2004 (4)	0.4110 (3)	0.2388 (2)	0.0339 (16)	
O21	0.0657 (3)	0.6585 (2)	0.10650 (17)	0.0476 (11)	
O22	0.2050 (3)	0.0315 (2)	0.13403 (17)	0.0516 (11)	
O23	−0.0887 (4)	0.1058 (2)	0.0027 (2)	0.0589 (12)	
H1A	−0.03770	0.14420	−0.57460	0.0510*	
H1B	0.19710	0.14380	−0.57160	0.0710*	
H2A	0.40160	0.10420	−0.01720	0.0610*	
H2B	0.31890	0.12910	−0.46590	0.0710*	
H4A	−0.04920	0.11230	−0.37520	0.0630*	
H5A	−0.16430	0.12860	−0.48310	0.0640*	
H6A	0.27560	0.05030	−0.35280	0.0790*	
H6B	0.31060	0.15810	−0.32000	0.0790*	
H7A	0.07590	0.06580	−0.28610	0.0740*	
H7B	0.10490	0.17400	−0.25540	0.0740*	
H9A	0.43240	0.11960	−0.22370	0.0760*	
H10A	0.54020	0.11440	−0.10810	0.0750*	
H11A	0.16730	0.09640	−0.03140	0.0700*	
H12A	0.05520	0.10140	−0.14290	0.0720*	
H3B	0.41830	0.36670	−0.11660	0.0670*	
H4C	0.85640	0.39690	0.46290	0.0510*	
H13B	0.65170	0.39940	−0.09460	0.0840*	
H14B	0.76500	0.40650	0.02050	0.0730*	
H16A	0.39190	0.34390	0.08860	0.0650*	
H17A	0.28660	0.34350	−0.02830	0.0730*	
H18B	0.74930	0.42320	0.15630	0.0530*	
H18C	0.71200	0.31620	0.13300	0.0530*	
H19B	0.54230	0.45190	0.22220	0.0500*	
H19C	0.50670	0.34470	0.19950	0.0500*	
H21'	0.86060	0.38050	0.24750	0.0540*	
H22'	0.97830	0.38260	0.36130	0.0590*	
H23'	0.62350	0.41070	0.46300	0.0540*	
H24A	0.49900	0.41030	0.35150	0.0510*	
H3A	0.14950	0.61320	0.40470	0.0720*	
H4B	−0.17370	0.65030	0.55600	0.0730*	
H5B	−0.44890	0.67260	0.47540	0.0850*	
H27A	−0.00220	0.61420	0.29570	0.0460*	
H31A	−0.41270	0.64150	0.33840	0.0450*	
H8A	0.15350	0.77980	0.15950	0.0790*	
H9B	0.30390	1.05660	0.30550	0.0860*	
H10B	0.45880	1.06870	0.38110	0.0740*	
H34A	0.22580	0.71490	0.25220	0.0440*	
H38A	0.43660	0.89480	0.44760	0.0410*	
H13A	0.98090	0.14910	0.29170	0.0680*	
H14A	0.66750	0.20550	0.44280	0.0670*	
H15A	0.39310	0.23340	0.36030	0.0560*	
H41A	0.81870	0.11870	0.17310	0.0460*	
H45A	0.41840	0.17300	0.22140	0.0400*	
H18A	0.03020	0.23320	−0.00500	0.0770*	
H19A	0.14940	0.47830	0.05230	0.0770*	
H20A	0.15660	0.57970	0.20320	0.0760*	
H48A	0.05320	0.20960	0.12950	0.0440*	
H52A	0.24740	0.44200	0.28780	0.0410*	
H21A	0.00960	0.66790	0.14310	0.0710*	
H21B	0.02260	0.61940	0.06440	0.0710*	
H22A	0.23900	0.01750	0.17290	0.0780*	
H22B	0.27470	0.05590	0.11760	0.0780*	
H23A	−0.16510	0.11150	0.02650	0.0880*	
H23B	−0.11830	0.06250	−0.04050	0.0880*	

### Atomic displacement parameters (Å^2^)

**Table:**

	*U*^11^	*U*^22^	*U*^33^	*U*^12^	*U*^13^	*U*^23^
N1	0.042 (2)	0.060 (3)	0.032 (2)	0.009 (2)	−0.0031 (18)	0.023 (2)
N2	0.056 (3)	0.070 (3)	0.028 (2)	0.016 (2)	−0.007 (2)	0.018 (2)
C1	0.043 (3)	0.096 (4)	0.049 (3)	0.011 (3)	0.006 (3)	0.039 (3)
C2	0.032 (3)	0.107 (5)	0.049 (3)	0.012 (3)	0.002 (2)	0.040 (3)
C3	0.046 (3)	0.058 (3)	0.028 (3)	0.013 (2)	−0.002 (2)	0.016 (2)
C4	0.046 (3)	0.084 (4)	0.036 (3)	0.017 (3)	0.008 (2)	0.028 (3)
C5	0.044 (3)	0.081 (4)	0.042 (3)	0.015 (3)	−0.002 (3)	0.028 (3)
C6	0.062 (4)	0.101 (5)	0.042 (3)	0.030 (3)	−0.004 (3)	0.031 (3)
C7	0.067 (4)	0.090 (4)	0.035 (3)	0.016 (3)	−0.005 (3)	0.032 (3)
C8	0.053 (3)	0.054 (3)	0.037 (3)	0.009 (3)	−0.008 (2)	0.018 (2)
C9	0.054 (4)	0.104 (5)	0.043 (3)	0.016 (3)	0.009 (3)	0.037 (3)
C10	0.051 (3)	0.092 (5)	0.049 (3)	0.011 (3)	0.002 (3)	0.030 (3)
C11	0.056 (4)	0.082 (4)	0.037 (3)	0.018 (3)	0.002 (3)	0.021 (3)
C12	0.055 (3)	0.094 (4)	0.041 (3)	0.019 (3)	0.003 (3)	0.034 (3)
N3	0.052 (3)	0.089 (4)	0.031 (2)	0.014 (2)	−0.007 (2)	0.027 (2)
N4	0.054 (3)	0.047 (2)	0.027 (2)	0.000 (2)	−0.0111 (19)	0.0169 (18)
C13	0.055 (4)	0.123 (6)	0.044 (3)	0.008 (3)	−0.003 (3)	0.045 (3)
C14	0.032 (3)	0.108 (5)	0.048 (3)	0.000 (3)	−0.003 (2)	0.037 (3)
C15	0.039 (3)	0.052 (3)	0.033 (3)	0.005 (2)	0.000 (2)	0.017 (2)
C16	0.046 (3)	0.087 (4)	0.036 (3)	0.005 (3)	−0.001 (2)	0.030 (3)
C17	0.036 (3)	0.108 (5)	0.038 (3)	0.014 (3)	−0.005 (2)	0.027 (3)
C18	0.035 (3)	0.072 (4)	0.032 (3)	0.007 (2)	−0.006 (2)	0.026 (2)
C19	0.034 (3)	0.059 (3)	0.035 (3)	0.008 (2)	−0.007 (2)	0.020 (2)
C20	0.038 (3)	0.036 (3)	0.028 (2)	0.003 (2)	−0.004 (2)	0.007 (2)
C21	0.046 (3)	0.061 (3)	0.033 (3)	0.010 (3)	−0.001 (2)	0.022 (2)
C22	0.052 (3)	0.061 (3)	0.034 (3)	0.008 (3)	−0.007 (2)	0.019 (3)
C23	0.048 (3)	0.047 (3)	0.040 (3)	−0.007 (2)	0.003 (2)	0.018 (2)
C24	0.040 (3)	0.054 (3)	0.034 (3)	0.000 (2)	−0.001 (2)	0.017 (2)
O1	0.054 (2)	0.160 (4)	0.038 (2)	0.025 (3)	0.0006 (18)	0.051 (2)
O2	0.052 (2)	0.163 (4)	0.044 (2)	0.007 (3)	0.0105 (19)	0.058 (3)
O3	0.0293 (18)	0.084 (3)	0.0347 (18)	0.0022 (17)	−0.0039 (14)	0.0272 (18)
O4	0.049 (2)	0.078 (2)	0.0246 (17)	0.0121 (18)	−0.0006 (15)	0.0246 (17)
O5	0.048 (2)	0.096 (3)	0.037 (2)	0.0267 (19)	0.0122 (16)	0.0341 (19)
C25	0.047 (3)	0.057 (3)	0.033 (3)	0.005 (2)	0.000 (2)	0.022 (2)
C26	0.039 (3)	0.042 (3)	0.024 (2)	0.001 (2)	−0.004 (2)	0.012 (2)
C27	0.037 (3)	0.053 (3)	0.030 (3)	0.002 (2)	0.005 (2)	0.022 (2)
C28	0.031 (2)	0.043 (3)	0.031 (3)	0.006 (2)	−0.005 (2)	0.014 (2)
C29	0.045 (3)	0.039 (3)	0.023 (2)	0.007 (2)	−0.004 (2)	0.012 (2)
C30	0.039 (3)	0.049 (3)	0.030 (3)	0.007 (2)	0.001 (2)	0.018 (2)
C31	0.034 (3)	0.055 (3)	0.024 (2)	0.008 (2)	−0.003 (2)	0.014 (2)
O6	0.059 (2)	0.048 (2)	0.0269 (18)	0.0137 (16)	−0.0055 (15)	0.0139 (15)
O7	0.053 (2)	0.040 (2)	0.0322 (17)	0.0013 (16)	−0.0031 (15)	0.0168 (15)
O8	0.075 (2)	0.049 (2)	0.0322 (18)	−0.0060 (18)	−0.0190 (17)	0.0188 (16)
O9	0.090 (3)	0.0366 (19)	0.047 (2)	−0.0062 (18)	−0.0157 (19)	0.0209 (17)
O10	0.069 (2)	0.041 (2)	0.0363 (18)	−0.0134 (17)	−0.0082 (16)	0.0162 (16)
C32	0.037 (3)	0.042 (3)	0.029 (3)	0.010 (2)	0.002 (2)	0.014 (2)
C33	0.031 (2)	0.039 (3)	0.030 (2)	0.004 (2)	−0.002 (2)	0.013 (2)
C34	0.039 (3)	0.036 (3)	0.032 (3)	0.001 (2)	−0.007 (2)	0.010 (2)
C35	0.042 (3)	0.043 (3)	0.029 (3)	0.007 (2)	−0.007 (2)	0.014 (2)
C36	0.038 (3)	0.040 (3)	0.035 (3)	0.000 (2)	−0.001 (2)	0.018 (2)
C37	0.040 (3)	0.037 (3)	0.031 (3)	−0.001 (2)	0.000 (2)	0.013 (2)
C38	0.030 (2)	0.047 (3)	0.026 (2)	0.006 (2)	−0.0007 (19)	0.013 (2)
O11	0.046 (2)	0.090 (3)	0.0287 (18)	0.0067 (19)	−0.0037 (15)	0.0224 (18)
O12	0.055 (2)	0.130 (4)	0.038 (2)	0.005 (2)	0.0115 (18)	0.032 (2)
O13	0.0320 (18)	0.072 (2)	0.0417 (19)	0.0050 (16)	0.0023 (14)	0.0317 (18)
O14	0.0412 (18)	0.064 (2)	0.0291 (18)	0.0093 (16)	−0.0045 (14)	0.0169 (16)
O15	0.0407 (18)	0.0471 (19)	0.0261 (16)	0.0080 (15)	0.0012 (14)	0.0153 (14)
C39	0.042 (3)	0.055 (3)	0.033 (3)	0.006 (2)	0.001 (2)	0.016 (2)
C40	0.041 (3)	0.035 (3)	0.029 (2)	0.005 (2)	0.001 (2)	0.009 (2)
C41	0.037 (3)	0.054 (3)	0.024 (2)	0.006 (2)	0.004 (2)	0.015 (2)
C42	0.030 (2)	0.040 (3)	0.041 (3)	−0.001 (2)	−0.002 (2)	0.018 (2)
C43	0.040 (3)	0.037 (3)	0.022 (2)	−0.002 (2)	−0.006 (2)	0.013 (2)
C44	0.039 (3)	0.036 (3)	0.021 (2)	0.004 (2)	−0.0001 (19)	0.0104 (19)
C45	0.033 (2)	0.037 (3)	0.032 (3)	0.004 (2)	−0.001 (2)	0.014 (2)
O16	0.054 (2)	0.052 (2)	0.0302 (18)	0.0075 (16)	−0.0033 (15)	0.0175 (16)
O17	0.045 (2)	0.045 (2)	0.0454 (19)	0.0023 (16)	0.0053 (15)	0.0200 (16)
O18	0.077 (2)	0.0431 (19)	0.0268 (17)	−0.0013 (17)	−0.0189 (16)	0.0086 (15)
O19	0.069 (2)	0.053 (2)	0.0364 (19)	−0.0007 (18)	−0.0103 (16)	0.0239 (17)
O20	0.075 (2)	0.0326 (19)	0.043 (2)	−0.0025 (17)	−0.0101 (17)	0.0156 (15)
C46	0.037 (3)	0.041 (3)	0.030 (3)	0.012 (2)	0.007 (2)	0.018 (2)
C47	0.028 (2)	0.038 (3)	0.024 (2)	0.007 (2)	−0.0002 (18)	0.009 (2)
C48	0.044 (3)	0.034 (3)	0.031 (2)	−0.001 (2)	−0.004 (2)	0.012 (2)
C49	0.041 (3)	0.039 (3)	0.022 (2)	0.002 (2)	−0.010 (2)	0.008 (2)
C50	0.044 (3)	0.040 (3)	0.030 (3)	0.002 (2)	−0.002 (2)	0.016 (2)
C51	0.045 (3)	0.033 (3)	0.032 (3)	0.003 (2)	0.001 (2)	0.012 (2)
C52	0.036 (3)	0.039 (3)	0.026 (2)	0.005 (2)	−0.0030 (19)	0.011 (2)
O21	0.052 (2)	0.060 (2)	0.0297 (17)	−0.0049 (17)	0.0001 (15)	0.0159 (16)
O22	0.062 (2)	0.053 (2)	0.0400 (19)	−0.0076 (17)	−0.0063 (16)	0.0198 (17)
O23	0.055 (2)	0.056 (2)	0.051 (2)	−0.0081 (18)	0.0041 (17)	0.0012 (18)

### Geometric parameters (Å, °)

**Table:**

O1—C25	1.271 (6)	C6—H6B	0.9700
O2—C25	1.220 (6)	C7—H7A	0.9700
O3—C28	1.367 (5)	C7—H7B	0.9700
O4—C29	1.355 (5)	C9—H9A	0.9300
O5—C30	1.375 (5)	C10—H10A	0.9300
O3—H3A	0.8200	C11—H11A	0.9300
O4—H4B	0.8200	C12—H12A	0.9300
O5—H5B	0.8200	C13—C14	1.357 (8)
O6—C32	1.265 (6)	C14—C15	1.380 (7)
O7—C32	1.258 (6)	C15—C18	1.507 (6)
O8—C35	1.369 (5)	C15—C16	1.381 (7)
O9—C36	1.356 (6)	C16—C17	1.383 (7)
O10—C37	1.371 (5)	C18—C19	1.518 (6)
O8—H8A	0.8200	C19—C20	1.495 (6)
O9—H9B	0.8200	C20—C21	1.379 (7)
O10—H10B	0.8200	C20—C24	1.384 (6)
O11—C39	1.272 (6)	C21—C22	1.366 (7)
O12—C39	1.216 (6)	C23—C24	1.361 (7)
O13—C42	1.388 (5)	C13—H13B	0.9300
O14—C43	1.359 (5)	C14—H14B	0.9300
O15—C44	1.374 (5)	C16—H16A	0.9300
O13—H13A	0.8200	C17—H17A	0.9300
O14—H14A	0.8200	C18—H18B	0.9700
O15—H15A	0.8200	C18—H18C	0.9700
N1—C1	1.331 (6)	C19—H19B	0.9700
N1—C5	1.308 (6)	C19—H19C	0.9700
N2—C10	1.328 (7)	C21—H21'	0.9300
N2—C11	1.324 (7)	C22—H22'	0.9300
O16—C46	1.260 (6)	C23—H23'	0.9300
O17—C46	1.269 (6)	C24—H24A	0.9300
O18—C49	1.362 (5)	C25—C26	1.497 (6)
O19—C50	1.367 (5)	C26—C31	1.383 (6)
O20—C51	1.362 (6)	C26—C27	1.385 (7)
N1—H1A	0.8600	C27—C28	1.390 (5)
N2—H2A	0.8600	C28—C29	1.389 (6)
O18—H18A	0.8200	C29—C30	1.390 (7)
O19—H19A	0.8200	C30—C31	1.382 (5)
O20—H20A	0.8200	C27—H27A	0.9300
N3—C13	1.326 (7)	C31—H31A	0.9300
N3—C17	1.324 (7)	C32—C33	1.496 (7)
N4—C23	1.335 (6)	C33—C34	1.403 (5)
N4—C22	1.319 (6)	C33—C38	1.382 (6)
N3—H3B	0.8600	C34—C35	1.385 (7)
N4—H4C	0.8600	C35—C36	1.391 (7)
O21—H21A	0.8600	C36—C37	1.381 (6)
O21—H21B	0.8600	C37—C38	1.382 (6)
O22—H22B	0.8600	C34—H34A	0.9300
O22—H22A	0.8600	C38—H38A	0.9300
O23—H23A	0.8600	C39—C40	1.505 (6)
O23—H23B	0.8600	C40—C41	1.389 (7)
C1—C2	1.347 (7)	C40—C45	1.379 (6)
C2—C3	1.393 (7)	C41—C42	1.369 (6)
C3—C4	1.374 (7)	C42—C43	1.391 (6)
C3—C6	1.517 (8)	C43—C44	1.386 (6)
C4—C5	1.366 (8)	C44—C45	1.381 (5)
C6—C7	1.482 (8)	C41—H41A	0.9300
C7—C8	1.515 (7)	C45—H45A	0.9300
C8—C9	1.370 (7)	C46—C47	1.492 (7)
C8—C12	1.383 (7)	C47—C48	1.390 (5)
C9—C10	1.390 (8)	C47—C52	1.392 (7)
C11—C12	1.348 (8)	C48—C49	1.379 (6)
C1—H1B	0.9300	C49—C50	1.400 (7)
C2—H2B	0.9300	C50—C51	1.390 (6)
C4—H4A	0.9300	C51—C52	1.380 (7)
C5—H5A	0.9300	C48—H48A	0.9300
C6—H6A	0.9700	C52—H52A	0.9300
			
O1···C13^i^	3.249 (7)	C16···H19C	2.7800
O1···N3^i^	2.514 (5)	C16···H19B	3.0100
O2···O21	2.680 (5)	C17···H19A	3.0500
O3···C32	3.217 (6)	C18···H21'	2.5200
O3···O7	2.652 (4)	C19···H16A	2.6800
O3···O4	2.637 (4)	C21···H18C	2.7900
O3···N4^ii^	2.804 (5)	C21···H18B	2.7400
O4···O3	2.637 (4)	C25···H18B^iv^	3.0000
O4···O5	2.680 (4)	C25···H3B^i^	2.4900
O4···C22^ii^	2.996 (6)	C25···H21A	3.0600
O4···N4^ii^	3.026 (5)	C25···H7B^i^	3.0700
O4···O16^iii^	2.804 (4)	C28···H4C^ii^	2.9900
O5···O6^iv^	2.634 (5)	C32···H5A^i^	3.0800
O5···O4	2.680 (4)	C32···H3A	2.4800
O5···O15^iii^	3.027 (5)	C32···H31A^v^	2.6800
O6···O5^v^	2.634 (5)	C32···H14A^ii^	2.9100
O6···O14^ii^	2.698 (4)	C32···H5B^v^	2.6800
O6···O15^ii^	3.121 (4)	C33···H5A^i^	2.8800
O6···C31^v^	3.409 (5)	C34···H20A	2.9300
O6···C30^v^	3.349 (6)	C34···H4A^i^	2.9900
O7···C52	3.400 (5)	C35···H4A^i^	3.0100
O7···O3	2.652 (4)	C36···H22A^vi^	2.9700
O7···O20	2.608 (4)	C38···H5A^i^	2.7600
O7···C51	3.370 (6)	C39···H2A	2.4900
O8···O21	2.715 (4)	C39···H18C	3.0100
O8···O9	2.735 (5)	C39···H22B	2.9900
O9···O15^vi^	3.153 (4)	C45···H22B	3.0200
O9···C45^vi^	3.266 (6)	C45···H19C	3.0500
O9···O22^vi^	2.713 (4)	C46···H22'^iv^	2.8800
O9···O10	2.712 (4)	C46···H4B^iii^	2.9800
O9···O8	2.735 (5)	C46···H45A	2.7700
O9···C44^vi^	3.254 (6)	C46···H15A	2.6700
O10···C44^vi^	3.334 (5)	C47···H21'^iv^	3.0100
O10···C1^vii^	3.280 (6)	C48···H21'^iv^	3.0900
O10···C2^vii^	3.157 (6)	C50···H16A	2.9800
O10···O15^vi^	2.812 (5)	C51···H16A	3.0300
O10···O9	2.712 (4)	H1A···H13A^ix^	2.4900
O11···N2	2.571 (5)	H1A···O13^ix^	1.9800
O11···O22	2.855 (5)	H1A···C43^ix^	3.0700
O11···C11	3.256 (6)	H1A···O14^ix^	2.3200
O13···O14	2.609 (4)	H1A···C42^ix^	2.9700
O13···C46^v^	3.410 (6)	H1B···H9B^x^	2.5000
O13···O17^v^	2.569 (4)	H1B···O10^x^	2.8400
O14···O15	2.729 (4)	H1B···O15^xii^	2.9100
O14···O13	2.609 (4)	H2A···O11	1.7400
O15···O5^iii^	3.027 (5)	H2A···O12	2.6000
O15···O14	2.729 (4)	H2A···C39	2.4900
O15···O16	2.713 (4)	H2B···O10^x^	2.5900
O16···O4^iii^	2.804 (4)	H2B···H38A^xi^	2.3800
O16···O15	2.713 (4)	H2B···H6B	2.5600
O17···O22	2.717 (4)	H2B···C38^xi^	2.9700
O18···O19	2.647 (5)	H3A···H27A	2.3900
O18···O23	2.623 (5)	H3A···H4C^ii^	2.4800
O19···O21	2.916 (5)	H3A···O7	1.8300
O19···O20	2.721 (4)	H3A···C32	2.4800
O19···O18	2.647 (5)	H3B···O1^i^	1.6700
O20···C34	3.265 (6)	H3B···C25^i^	2.4900
O20···O19	2.721 (4)	H3B···O2^i^	2.6800
O20···O7	2.608 (4)	H4A···C7	2.6000
O20···O21	3.185 (5)	H4A···C35^i^	3.0100
O21···O2	2.680 (5)	H4A···H7A	2.2800
O21···O19	2.916 (5)	H4A···H7B	2.4200
O21···O8	2.715 (4)	H4A···C34^i^	2.9900
O21···O20	3.185 (5)	H4B···O5	2.2900
O22···O17	2.717 (4)	H4B···C46^iii^	2.9800
O22···O11	2.855 (5)	H4B···O16^iii^	2.0600
O23···C48	3.225 (5)	H4B···H22'^ii^	2.4900
O23···C49	3.297 (6)	H4C···O4^ii^	2.4700
O23···O18	2.623 (5)	H4C···H3A^ii^	2.4800
O23···C11	3.293 (7)	H4C···O3^ii^	1.9700
O1···H3B^i^	1.6700	H4C···C28^ii^	2.9900
O1···H31A	2.4100	H5A···C38^i^	2.7600
O2···H7B^i^	2.9100	H5A···C32^i^	3.0800
O2···H21B	2.7700	H5A···H14A^ix^	2.6000
O2···H3B^i^	2.6800	H5A···C33^i^	2.8800
O2···H21A	1.9200	H5A···O14^ix^	2.4400
O2···H27A	2.5600	H5B···O15^iii^	2.8000
O3···H4C^ii^	1.9700	H5B···C32^iv^	2.6800
O4···H22'^ii^	2.3800	H5B···H23'^iii^	2.5700
O4···H4C^ii^	2.4700	H5B···O6^iv^	1.8100
O5···H4B	2.2900	H5B···H31A	2.4400
O5···H15A^iii^	2.8500	H6A···C9	2.9700
O5···H23'^iii^	2.7200	H6A···H9A	2.5700
O6···H31A^v^	2.8900	H6B···H2B	2.5600
O6···H38A	2.4500	H6B···H9A	2.3000
O6···H5B^v^	1.8100	H6B···C9	2.8100
O6···H14A^ii^	1.9400	H7A···C4	2.7900
O7···H3A	1.8300	H7A···O13^xiii^	2.9000
O7···H52A	2.8000	H7A···H4A	2.2800
O7···H31A^v^	2.7800	H7A···H12A	2.5100
O7···H27A	2.8800	H7B···H4A	2.4200
O7···H20A	2.7500	H7B···C4	2.8300
O7···H34A	2.5500	H7B···O2^i^	2.9100
O8···H22A^vi^	2.8800	H7B···C25^i^	3.0700
O8···H12A^i^	2.7000	H8A···O21	1.9000
O8···H21A	2.8400	H8A···H34A	2.3200
O9···H10B	2.3400	H8A···H21A	2.0600
O9···H22A^vi^	1.8600	H9A···H6B	2.3000
O10···H2B^vii^	2.5900	H9A···H6A	2.5700
O10···H1B^vii^	2.8400	H9A···C35^xi^	3.0700
O10···H9B	2.3400	H9A···C36^xi^	2.8500
O10···H38A^viii^	2.6100	H9A···C6	2.6700
O11···H2A	1.7400	H9B···O15^vi^	2.4700
O11···H22B	1.9900	H9B···H22A^vi^	2.3300
O11···H45A	2.4600	H9B···H10B	1.9000
O12···H41A	2.6300	H9B···O10	2.3400
O12···H18C	2.8300	H9B···H1B^vii^	2.5000
O12···H2A	2.6000	H9B···C45^vi^	3.0900
O15···H5B^iii^	2.8000	H9B···C44^vi^	2.8500
O15···H14A	2.3200	H10B···C43^vi^	2.8400
O16···H52A	2.5400	H10B···C44^vi^	2.5300
O16···H22'^iv^	2.9000	H10B···O9	2.3400
O16···H24A	2.7100	H10B···H9B	1.9000
O16···H4B^iii^	2.0600	H10B···O15^vi^	2.1200
O16···H15A	1.8900	H10B···O14^vi^	2.8800
O17···H22A	2.7300	H11A···O23	2.5300
O17···H48A	2.4600	H11A···H18A	2.4400
O17···H45A	2.8300	H12A···H7A	2.5100
O19···H20A	2.6500	H12A···O8^i^	2.7000
O19···H21B	2.5400	H13A···H41A	2.4700
O20···H19A	2.6200	H13A···C46^v^	2.6500
O20···H34A	2.4000	H13A···O17^v^	1.7500
O20···H27A	2.8300	H13B···H19A^xi^	2.4700
O21···H34A	2.8200	H13B···O21^xi^	2.8500
O21···H8A	1.9000	H13B···O20^xi^	2.6800
O21···H20A	2.5900	H14A···O15	2.3200
O21···H19A	2.7700	H14B···H18B	2.4300
O22···H45A	2.8500	H14B···H21B^xi^	2.5800
O23···H11A	2.5300	H14B···O21^xi^	2.8300
O23···H48A	2.5700	H15A···H45A	2.4200
O23···H18A	2.2500	H15A···O5^iii^	2.8500
N1···O13^ix^	2.793 (5)	H15A···C46	2.6700
N1···O14^ix^	2.914 (5)	H15A···O16	1.8900
N2···O11	2.571 (5)	H16A···C51	3.0300
N2···C39	3.326 (6)	H16A···C50	2.9800
N3···C25^i^	3.335 (6)	H16A···C19	2.6800
N3···O1^i^	2.514 (5)	H16A···H19C	2.2400
N4···C29^v^	3.344 (7)	H17A···H19A	2.5600
N4···C43	3.442 (6)	H18A···O23	2.2500
N4···O3^ii^	2.804 (5)	H18A···H11A	2.4400
N4···O4^ii^	3.026 (5)	H18A···C12	2.9100
C1···O13^ix^	3.414 (6)	H18A···C11	2.7900
C1···O10^x^	3.280 (6)	H18B···H14B	2.4300
C1···C30^i^	3.490 (8)	H18B···H21'	2.2200
C1···C29^i^	3.424 (8)	H18B···C21	2.7400
C2···C30^i^	3.471 (8)	H18B···C25^v^	3.0000
C2···O10^x^	3.157 (6)	H18C···H21'	2.3300
C2···C31^i^	3.538 (7)	H18C···C39	3.0100
C5···O14^ix^	2.984 (6)	H18C···C21	2.7900
C5···C38^i^	3.522 (6)	H18C···O12	2.8300
C5···C33^i^	3.502 (6)	H19A···H21B	2.4800
C11···O11	3.256 (6)	H19A···O21	2.7700
C11···O23	3.293 (7)	H19A···O20	2.6200
C13···O21^xi^	3.395 (6)	H19A···H17A	2.5600
C13···O1^i^	3.249 (7)	H19A···C17	3.0500
C14···O21^xi^	3.375 (6)	H19B···C16	3.0100
C16···C50	3.560 (7)	H19C···C45	3.0500
C20···C31^v^	3.468 (7)	H19C···C16	2.7800
C20···C45	3.531 (7)	H19C···H16A	2.2400
C21···C27^v^	3.488 (7)	H20A···O21	2.5900
C21···C28^v^	3.564 (7)	H20A···H34A	2.0100
C22···C28^v^	3.331 (7)	H20A···O7	2.7500
C22···C42	3.567 (7)	H20A···H27A	2.2800
C22···C29^v^	3.494 (7)	H20A···C34	2.9300
C22···O4^ii^	2.996 (6)	H20A···H21A	2.4400
C23···C30^v^	3.465 (7)	H20A···O19	2.6500
C23···C29^v^	3.542 (7)	H21'···C48^v^	3.0900
C23···C43	3.454 (7)	H21'···H18C	2.3300
C23···C44	3.433 (7)	H21'···C18	2.5200
C24···C44	3.289 (7)	H21'···C47^v^	3.0100
C24···C45	3.564 (7)	H21'···H18B	2.2200
C24···C31^v^	3.584 (7)	H21A···O8	2.8400
C24···C30^v^	3.419 (7)	H21A···O2	1.9200
C25···N3^i^	3.335 (6)	H21A···C25	3.0600
C27···C21^iv^	3.488 (7)	H21A···H20A	2.4400
C28···C21^iv^	3.564 (7)	H21A···H8A	2.0600
C28···C22^iv^	3.331 (7)	H21B···O2	2.7700
C29···C22^iv^	3.494 (7)	H21B···H19A	2.4800
C29···C23^iv^	3.542 (7)	H21B···O19	2.5400
C29···C1^i^	3.424 (8)	H22'···H4B^ii^	2.4900
C29···N4^iv^	3.344 (7)	H22'···O4^ii^	2.3800
C30···C24^iv^	3.419 (7)	H22'···C46^v^	2.8800
C30···C23^iv^	3.465 (7)	H22'···O16^v^	2.9000
C30···C2^i^	3.471 (8)	H22A···O17	2.7300
C30···C1^i^	3.490 (8)	H22B···C39	2.9900
C30···O6^iv^	3.349 (6)	H22B···H45A	2.4000
C31···C32^iv^	3.467 (7)	H22B···O11	1.9900
C31···C2^i^	3.538 (7)	H22B···C45	3.0200
C31···C24^iv^	3.584 (7)	H23'···O5^iii^	2.7200
C31···O6^iv^	3.409 (5)	H23'···H5B^iii^	2.5700
C31···C20^iv^	3.468 (7)	H24A···O16	2.7100
C32···C31^v^	3.467 (7)	H27A···O20	2.8300
C32···O3	3.217 (6)	H27A···H3A	2.3900
C33···C5^i^	3.502 (6)	H27A···O7	2.8800
C34···O20	3.265 (6)	H27A···O2	2.5600
C38···C5^i^	3.522 (6)	H27A···H20A	2.2800
C39···N2	3.326 (6)	H31A···O1	2.4100
C42···C22	3.567 (7)	H31A···H5B	2.4400
C42···O17^v^	3.393 (5)	H31A···C32^iv^	2.6800
C43···C23	3.454 (7)	H31A···O6^iv^	2.8900
C43···N4	3.442 (6)	H31A···O7^iv^	2.7800
C44···C23	3.433 (7)	H34A···O20	2.4000
C44···C24	3.289 (7)	H34A···O21	2.8200
C45···C46	3.552 (7)	H34A···H20A	2.0100
C45···C24	3.564 (7)	H34A···H8A	2.3200
C45···C20	3.531 (7)	H34A···O7	2.5500
C46···C45	3.552 (7)	H38A···O10^viii^	2.6100
C48···O23	3.225 (5)	H38A···O6	2.4500
C49···O23	3.297 (6)	H41A···O12	2.6300
C50···C16	3.560 (7)	H41A···H13A	2.4700
C51···O7	3.370 (6)	H45A···O17	2.8300
C52···O7	3.400 (5)	H45A···O22	2.8500
C4···H7B	2.8300	H45A···C46	2.7700
C4···H7A	2.7900	H45A···H15A	2.4200
C6···H9A	2.6700	H45A···H22B	2.4000
C7···H4A	2.6000	H45A···O11	2.4600
C9···H6B	2.8100	H48A···O17	2.4600
C9···H6A	2.9700	H48A···O23	2.5700
C11···H18A	2.7900	H52A···O7	2.8000
C12···H18A	2.9100	H52A···O16	2.5400
C13···H19A^xi^	3.0300		
			
C28—O3—H3A	110	C18—C19—H19C	109
C29—O4—H4B	109	C20—C19—H19B	109
C30—O5—H5B	109	H19B—C19—H19C	108
C35—O8—H8A	109	C20—C19—H19C	109
C36—O9—H9B	110	C18—C19—H19B	109
C37—O10—H10B	109	C22—C21—H21'	120
C42—O13—H13A	110	C20—C21—H21'	120
C43—O14—H14A	110	N4—C22—H22'	120
C44—O15—H15A	109	C21—C22—H22'	120
C1—N1—C5	121.8 (4)	N4—C23—H23'	120
C10—N2—C11	120.7 (4)	C24—C23—H23'	120
C1—N1—H1A	119	C23—C24—H24A	120
C5—N1—H1A	119	C20—C24—H24A	120
C11—N2—H2A	120	O1—C25—C26	115.4 (4)
C10—N2—H2A	120	O1—C25—O2	122.4 (5)
C49—O18—H18A	109	O2—C25—C26	122.2 (4)
C50—O19—H19A	109	C25—C26—C27	119.5 (4)
C51—O20—H20A	110	C25—C26—C31	119.5 (4)
C13—N3—C17	118.5 (4)	C27—C26—C31	121.0 (4)
C22—N4—C23	121.1 (4)	C26—C27—C28	119.2 (4)
C17—N3—H3B	121	C27—C28—C29	120.4 (4)
C13—N3—H3B	121	O3—C28—C29	115.6 (3)
C22—N4—H4C	119	O3—C28—C27	124.0 (4)
C23—N4—H4C	119	C28—C29—C30	119.4 (3)
H21A—O21—H21B	109	O4—C29—C30	121.6 (4)
H22A—O22—H22B	107	O4—C29—C28	119.0 (4)
H23A—O23—H23B	100	C29—C30—C31	120.7 (4)
N1—C1—C2	120.1 (5)	O5—C30—C31	124.5 (4)
C1—C2—C3	121.0 (5)	O5—C30—C29	114.9 (3)
C2—C3—C6	119.6 (4)	C26—C31—C30	119.3 (4)
C2—C3—C4	116.1 (5)	C26—C27—H27A	120
C4—C3—C6	124.3 (5)	C28—C27—H27A	120
C3—C4—C5	121.2 (5)	C26—C31—H31A	120
N1—C5—C4	120.0 (4)	C30—C31—H31A	120
C3—C6—C7	115.2 (5)	O6—C32—C33	117.3 (4)
C6—C7—C8	117.2 (5)	O7—C32—C33	119.7 (4)
C7—C8—C9	124.8 (5)	O6—C32—O7	123.0 (4)
C9—C8—C12	117.1 (5)	C32—C33—C38	119.6 (4)
C7—C8—C12	118.1 (5)	C34—C33—C38	119.8 (4)
C8—C9—C10	120.7 (5)	C32—C33—C34	120.5 (4)
N2—C10—C9	119.5 (5)	C33—C34—C35	119.4 (4)
N2—C11—C12	121.7 (5)	O8—C35—C36	116.6 (4)
C8—C12—C11	120.4 (5)	C34—C35—C36	120.5 (4)
C2—C1—H1B	120	O8—C35—C34	123.0 (4)
N1—C1—H1B	120	O9—C36—C37	117.9 (4)
C3—C2—H2B	120	C35—C36—C37	119.5 (4)
C1—C2—H2B	120	O9—C36—C35	122.6 (4)
C5—C4—H4A	119	C36—C37—C38	120.6 (4)
C3—C4—H4A	119	O10—C37—C38	118.8 (3)
C4—C5—H5A	120	O10—C37—C36	120.5 (4)
N1—C5—H5A	120	C33—C38—C37	120.1 (3)
C3—C6—H6A	109	C35—C34—H34A	120
H6A—C6—H6B	107	C33—C34—H34A	120
C7—C6—H6B	108	C33—C38—H38A	120
C3—C6—H6B	109	C37—C38—H38A	120
C7—C6—H6A	108	O11—C39—O12	122.0 (5)
C6—C7—H7A	108	O11—C39—C40	116.0 (4)
C6—C7—H7B	108	O12—C39—C40	121.9 (4)
C8—C7—H7B	108	C41—C40—C45	119.5 (3)
H7A—C7—H7B	107	C39—C40—C45	119.5 (4)
C8—C7—H7A	108	C39—C40—C41	121.0 (4)
C10—C9—H9A	120	C40—C41—C42	120.3 (4)
C8—C9—H9A	120	C41—C42—C43	120.2 (4)
C9—C10—H10A	120	O13—C42—C41	124.9 (4)
N2—C10—H10A	120	O13—C42—C43	114.9 (4)
N2—C11—H11A	119	C42—C43—C44	119.5 (4)
C12—C11—H11A	119	O14—C43—C44	122.7 (4)
C8—C12—H12A	120	O14—C43—C42	117.7 (4)
C11—C12—H12A	120	O15—C44—C45	124.0 (4)
N3—C13—C14	122.3 (5)	C43—C44—C45	119.8 (4)
C13—C14—C15	120.5 (5)	O15—C44—C43	116.2 (3)
C16—C15—C18	123.1 (5)	C40—C45—C44	120.5 (4)
C14—C15—C16	117.2 (5)	C42—C41—H41A	120
C14—C15—C18	119.7 (4)	C40—C41—H41A	120
C15—C16—C17	118.9 (5)	C40—C45—H45A	120
N3—C17—C16	122.6 (5)	C44—C45—H45A	120
C15—C18—C19	116.3 (4)	O16—C46—O17	123.0 (4)
C18—C19—C20	114.9 (4)	O16—C46—C47	119.4 (4)
C19—C20—C21	122.4 (4)	O17—C46—C47	117.6 (4)
C21—C20—C24	117.1 (4)	C46—C47—C52	120.8 (3)
C19—C20—C24	120.5 (4)	C48—C47—C52	119.3 (4)
C20—C21—C22	120.6 (4)	C46—C47—C48	119.9 (4)
N4—C22—C21	120.4 (4)	C47—C48—C49	121.3 (4)
N4—C23—C24	120.4 (5)	O18—C49—C48	124.3 (4)
C20—C24—C23	120.4 (4)	O18—C49—C50	116.6 (4)
C14—C13—H13B	119	C48—C49—C50	119.1 (3)
N3—C13—H13B	119	C49—C50—C51	119.7 (4)
C13—C14—H14B	120	O19—C50—C49	117.8 (3)
C15—C14—H14B	120	O19—C50—C51	122.5 (4)
C17—C16—H16A	120	O20—C51—C50	115.9 (4)
C15—C16—H16A	121	O20—C51—C52	123.5 (4)
C16—C17—H17A	119	C50—C51—C52	120.6 (4)
N3—C17—H17A	119	C47—C52—C51	120.0 (4)
C19—C18—H18C	108	C47—C48—H48A	119
C15—C18—H18B	108	C49—C48—H48A	119
C19—C18—H18B	108	C47—C52—H52A	120
C15—C18—H18C	108	C51—C52—H52A	120
H18B—C18—H18C	107		
			
C5—N1—C1—C2	−0.6 (9)	C29—C30—C31—C26	−0.9 (7)
C1—N1—C5—C4	0.3 (9)	O6—C32—C33—C34	−178.5 (4)
C10—N2—C11—C12	−0.6 (9)	O6—C32—C33—C38	−0.2 (6)
C11—N2—C10—C9	0.6 (9)	O7—C32—C33—C34	1.6 (6)
C13—N3—C17—C16	−2.1 (9)	O7—C32—C33—C38	179.9 (4)
C17—N3—C13—C14	0.9 (10)	C32—C33—C34—C35	179.3 (4)
C23—N4—C22—C21	−0.3 (8)	C38—C33—C34—C35	1.1 (6)
C22—N4—C23—C24	0.0 (8)	C32—C33—C38—C37	−179.8 (4)
N1—C1—C2—C3	0.8 (9)	C34—C33—C38—C37	−1.5 (6)
C1—C2—C3—C6	−179.1 (6)	C33—C34—C35—O8	179.2 (4)
C1—C2—C3—C4	−0.6 (9)	C33—C34—C35—C36	−1.5 (6)
C4—C3—C6—C7	−9.0 (9)	O8—C35—C36—O9	0.6 (7)
C6—C3—C4—C5	178.7 (6)	O8—C35—C36—C37	−178.3 (4)
C2—C3—C6—C7	169.4 (6)	C34—C35—C36—O9	−178.8 (4)
C2—C3—C4—C5	0.3 (9)	C34—C35—C36—C37	2.4 (7)
C3—C4—C5—N1	−0.2 (9)	O9—C36—C37—O10	−0.6 (7)
C3—C6—C7—C8	−177.6 (5)	O9—C36—C37—C38	178.3 (4)
C6—C7—C8—C12	−171.0 (6)	C35—C36—C37—O10	178.3 (4)
C6—C7—C8—C9	9.3 (9)	C35—C36—C37—C38	−2.8 (7)
C7—C8—C9—C10	179.2 (6)	O10—C37—C38—C33	−178.7 (4)
C7—C8—C12—C11	−179.2 (6)	C36—C37—C38—C33	2.4 (6)
C12—C8—C9—C10	−0.5 (9)	O11—C39—C40—C41	164.2 (5)
C9—C8—C12—C11	0.5 (9)	O11—C39—C40—C45	−15.1 (7)
C8—C9—C10—N2	0.0 (9)	O12—C39—C40—C41	−16.3 (8)
N2—C11—C12—C8	0.0 (9)	O12—C39—C40—C45	164.5 (5)
N3—C13—C14—C15	1.1 (10)	C39—C40—C41—C42	−176.8 (5)
C13—C14—C15—C18	178.0 (6)	C45—C40—C41—C42	2.5 (7)
C13—C14—C15—C16	−1.8 (9)	C39—C40—C45—C44	178.2 (4)
C16—C15—C18—C19	−23.1 (8)	C41—C40—C45—C44	−1.1 (7)
C18—C15—C16—C17	−179.2 (5)	C40—C41—C42—O13	177.6 (4)
C14—C15—C18—C19	157.1 (5)	C40—C41—C42—C43	−1.7 (7)
C14—C15—C16—C17	0.6 (8)	O13—C42—C43—O14	1.9 (6)
C15—C16—C17—N3	1.4 (9)	O13—C42—C43—C44	−179.8 (4)
C15—C18—C19—C20	−179.2 (5)	C41—C42—C43—O14	−178.8 (4)
C18—C19—C20—C24	−175.8 (5)	C41—C42—C43—C44	−0.5 (7)
C18—C19—C20—C21	3.4 (8)	O14—C43—C44—O15	−1.0 (7)
C24—C20—C21—C22	0.0 (7)	O14—C43—C44—C45	−179.9 (4)
C19—C20—C21—C22	−179.2 (5)	C42—C43—C44—O15	−179.2 (4)
C19—C20—C24—C23	179.0 (5)	C42—C43—C44—C45	1.9 (7)
C21—C20—C24—C23	−0.3 (7)	O15—C44—C45—C40	−180.0 (4)
C20—C21—C22—N4	0.3 (8)	C43—C44—C45—C40	−1.1 (7)
N4—C23—C24—C20	0.3 (8)	O16—C46—C47—C48	175.2 (4)
O1—C25—C26—C27	176.5 (5)	O16—C46—C47—C52	−5.2 (6)
O1—C25—C26—C31	−1.7 (7)	O17—C46—C47—C48	−3.4 (6)
O2—C25—C26—C27	−2.2 (7)	O17—C46—C47—C52	176.2 (4)
O2—C25—C26—C31	179.5 (5)	C46—C47—C48—C49	179.7 (4)
C25—C26—C27—C28	−178.5 (4)	C52—C47—C48—C49	0.0 (6)
C31—C26—C27—C28	−0.3 (7)	C46—C47—C52—C51	−179.8 (4)
C25—C26—C31—C30	179.9 (4)	C48—C47—C52—C51	−0.2 (6)
C27—C26—C31—C30	1.6 (7)	C47—C48—C49—O18	−179.7 (4)
C26—C27—C28—O3	177.6 (4)	C47—C48—C49—C50	−0.7 (6)
C26—C27—C28—C29	−1.8 (7)	O18—C49—C50—O19	0.3 (6)
O3—C28—C29—O4	2.8 (6)	O18—C49—C50—C51	−179.4 (4)
O3—C28—C29—C30	−176.9 (4)	C48—C49—C50—O19	−178.8 (4)
C27—C28—C29—O4	−177.7 (4)	C48—C49—C50—C51	1.6 (6)
C27—C28—C29—C30	2.6 (7)	O19—C50—C51—O20	−3.9 (7)
O4—C29—C30—O5	−0.5 (7)	O19—C50—C51—C52	178.6 (4)
O4—C29—C30—C31	179.0 (4)	C49—C50—C51—O20	175.7 (4)
C28—C29—C30—O5	179.3 (4)	C49—C50—C51—C52	−1.7 (7)
C28—C29—C30—C31	−1.2 (7)	O20—C51—C52—C47	−176.2 (4)
O5—C30—C31—C26	178.6 (5)	C50—C51—C52—C47	1.1 (7)

Symmetry codes: (i) −*x*, −*y*+1, −*z*; (ii) −*x*+1, −*y*+1, −*z*+1; (iii) −*x*, −*y*+1, −*z*+1; (iv) *x*−1, *y*, *z*; (v) *x*+1, *y*, *z*; (vi) *x*, *y*+1, *z*; (vii) *x*, *y*+1, *z*+1; (viii) −*x*+1, −*y*+2, −*z*+1; (ix) *x*−1, *y*, *z*−1; (x) *x*, *y*−1, *z*−1; (xi) −*x*+1, −*y*+1, −*z*; (xii) *x*, *y*, *z*−1; (xiii) −*x*+1, −*y*, −*z*.

### Hydrogen-bond geometry (Å, °)

**Table:**

Cg6 and Cg8 are the centroids of the C33–C38 and C47–C52 rings, respectively.

**Table:**

*D*—H···*A*	*D*—H	H···*A*	*D*···*A*	*D*—H···*A*
N1—H1A···O13^ix^	0.86	1.98	2.793 (5)	158
N1—H1A···O14^ix^	0.86	2.32	2.914 (5)	127
N2—H2A···O11	0.86	1.74	2.571 (5)	163
N2—H2A···O12	0.86	2.60	3.297 (5)	139
N3—H3B···O1^i^	0.86	1.67	2.514 (5)	166
N4—H4C···O3^ii^	0.86	1.97	2.804 (5)	165
N4—H4C···O4^ii^	0.86	2.47	3.026 (5)	123
O3—H3A···O7	0.82	1.83	2.652 (4)	175
O4—H4B···O16^iii^	0.82	2.06	2.804 (4)	151
O5—H5B···O6^iv^	0.82	1.81	2.634 (5)	180
O8—H8A···O21	0.82	1.90	2.715 (4)	175
O9—H9B···O15^vi^	0.82	2.47	3.153 (4)	141
O10—H10B···O15^vi^	0.82	2.12	2.812 (5)	142
O13—H13A···O17^v^	0.82	1.75	2.569 (4)	172
O14—H14A···O15	0.82	2.32	2.729 (4)	111
O15—H15A···O16	0.82	1.89	2.713 (4)	179
O18—H18A···O23	0.82	2.25	2.623 (5)	108
O20—H20A···O21	0.82	2.59	3.185 (5)	131
O21—H21A···O2	0.86	1.92	2.680 (5)	148
O21—H21B···O19^i^	0.86	2.32	3.163 (4)	165
O22—H22A···O9^xiv^	0.86	1.86	2.713 (4)	170
O22—H22B···O11	0.86	1.99	2.855 (5)	179
O23—H23A···O12^iv^	0.86	1.80	2.637 (5)	166
O23—H23B···O22^xv^	0.86	1.92	2.781 (5)	174
C11—H11A···O23	0.93	2.53	3.293 (7)	139
C34—H34A···O20	0.93	2.40	3.265 (6)	154
C4—H4A···Cg6^i^	0.93	2.81	3.571 (5)	140
C16—H16A···Cg8	0.93	2.91	3.737 (5)	149

Symmetry codes: (ix) *x*−1, *y*, *z*−1; (i) −*x*, −*y*+1, −*z*; (ii) −*x*+1, −*y*+1, −*z*+1; (iii) −*x*, −*y*+1, −*z*+1; (iv) *x*−1, *y*, *z*; (vi) *x*, *y*+1, *z*; (v) *x*+1, *y*, *z*; (xiv) *x*, *y*−1, *z*; (xv) −*x*, −*y*, −*z*.
